# Analysis of Volatile Components in Different *Ophiocordyceps sinensis* and Insect Host Products

**DOI:** 10.3390/molecules25071603

**Published:** 2020-03-31

**Authors:** Xuehong Qiu, Li Cao, Richou Han

**Affiliations:** Guangdong Key Laboratory of Animal Conservation and Resource Utilization, Guangdong Public Laboratory of Wild Animal Conservation and Utilization, Guangdong Institute of Applied Biological Resources, Guangzhou 510260, Guangdong, China; xhqiu@126.com (X.Q.); caol@giabr.gd.cn (L.C.)

**Keywords:** *Ophiocordyceps sinensis*, *Thitarodes* hosts, Chinese cordyceps, volatile compounds, HS-SPME GC×GC-QTOFMS, partial least squares-discriminant analysis

## Abstract

The artificial production of *Ophiocordyceps* *sinensis* mycelia and fruiting bodies and the Chinese cordyceps has been established. However, the volatile components from these *O. sinensis* products are not fully identified. An efficient, convenient, and widely used approach based on headspace solid-phase microextraction (HS-SPME) combined with comprehensive two-dimensional gas chromatography and quadrupole time-of-flight mass spectrometry (GC×GC-QTOFMS) was developed for the extraction and the analysis of volatile compounds from three categories of 16 products, including *O. sinensis* fungus, *Thitarodes* hosts of *O. sinensis*, and the Chinese cordyceps. A total of 120 volatile components including 36 alkanes, 25 terpenes, 17 aromatic hydrocarbons, 10 ketones, 5 olefines, 5 alcohols, 3 phenols, and 19 other compounds were identified. The contents of these components varied greatly among the products but alkanes, especially 2,5,6-trimethyldecane, 2,3-dimethylundecane and 2,2,4,4-tetramethyloctane, are the dominant compounds in general. Three categories of volatile compounds were confirmed by partial least squares-discriminant analysis (PLS-DA). This study provided an ideal method for characterizing and distinguishing different *O. sinensis* and insect hosts-based products.

## 1. Introduction

The Chinese cordyceps, a parasitic *Ophiocordyceps sinensis* fungus-*Thitarodes*/*Hepialus* caterpillar complex endemic only at an elevation of 3000–5000 m in the Tibetan plateau, is a valuable health food and medicinal herb [1]. Modern pharmacological studies indicate that the Chinese cordyceps is good for human circulatory, immune, hematogenic, cardiovascular, respiratory, and glandular systems [2,3,4]. Due to the limited distribution, high cost, and over exploitation, the natural Chinese cordyceps is extremely expensive and not satisfactory for market demand [5]. Therefore, artificial cultivation is needed to satisfy the natural resource protection and human comsumption.

The artificial cultivation of *O. sinensis* fruiting bodies on rice media [6] and host caterpillar *Thitarodes* spp. [7,8] has been established. Mycelial products of *O. sinensis* fungus have also been manufactured by fermentation technology [9]. More excitingly, the success of cultivation on a large scale has been achieved recently in China [10,11].

Several main bioactive compounds were detected in natural and cultured Chinese cordyceps and fermented fungal products, such as nucleosides (adenosine and inosine), carbohydrates (mannitol, trehalose, and polysaccharides), sterols (ergosterol), and sphingolipids, etc. [12,13,14,15]. Some free fatty acids and sterols in natural and cultured Chinese cordyceps were determined by one-step derivatization and GC/MS [16].

From the mycelia of *O. sinensis* cultured with solid-state media and submerged fermentation, 51 volatile compounds were identified, and there is a great difference in the numbers of compounds in the two mycelia, but phenols, acids, and alkanes were the major classes of compounds, while butylated hydroxytoluene was the most abundant volatile compound in both mycelia [17]. The volatile components in several commercial fermentation products from mycelial strains isolated from natural Chinese cordyceps were also analyzed, and 5,6-Dihydro-6-pentyl-2*H*-pyran-2-one (massoia lactone) was discovered as the dominant component in the essential oils of Jinshuibao capsule volatiles, and fatty acids including palmitic acid (C16:0) and linoleic acid (C18:2) were also found to be major volatile compositions of the fermentation products [18]. It seems that products from different cultivation methods may exhibit different volatile components. However, a comprehensive volatile profiling from natural and artificially cultivated Chinese cordyceps is unknown.

The Green Analytical Chemistry technique of headspace solid-phase microextraction (HS-SPME) coupled with comprehensive two-dimensional gas chromatography time-of-flight mass spectrometry (GC×GC-TOFMS) proves to be a sensitive, accurate, efficient, and convenient approach for volatile compounds analysis [19,20,21,22,23,24]. HS-SPME can extract chemicals directly from sample headspace for volatile compounds analysis. It is well suitable for volatile sampling by the advantages of ease of automation, solvent-free procedure, high preconcentration capacity, little manipulation of the sample, and high cost-efficiency [19,20,21,24,25]. GC×GC is a powerful technique with high resolution and enhanced sensitivity for separating and analyzing complex samples [19,20,21,24,25,26,27]. In addition, with the strengths in accurate mass measurements and good sensitivity in full-scan acquisition mode of TOFMS, GC×GC-TOFMS becomes an increasingly popular analytical technique for characterization of the chemical compositions of biological samples [19,20,21,24,27]. Recently, the combination of HS-SPME and GC×GC-TOFMS has been applied to volatile analysis in many fields [19,20,21,24]. However, there is no report of the simultaneous analysis of volatile components in different *O. sinensis* and insect hosts-based products by this method. 

In the present work, HS-SPME and GC×GC-QTOFMS were employed to analyze volatile compounds from three categories of samples, including *O. sinensis* fungus, insect hosts of *O. sinensis*, and the Chinese cordyceps. Qualitative analysis was performed by comparing the mass spectra with the library and confirmed by their retention indices and fragmentation patterns. In addition, the three categories (*O. sinensis* fungus, *Thitarodes* hosts of *O. sinensis*, and the Chinese cordyceps) of samples were comparatively analyzed and differentiated using this method combined with multivariate partial least squares-discriminant analysis (PLS-DA).

## 2. Results and Discussion

### 2.1. Comparison of 1-DGC and GC×GC

**Figure 1:**
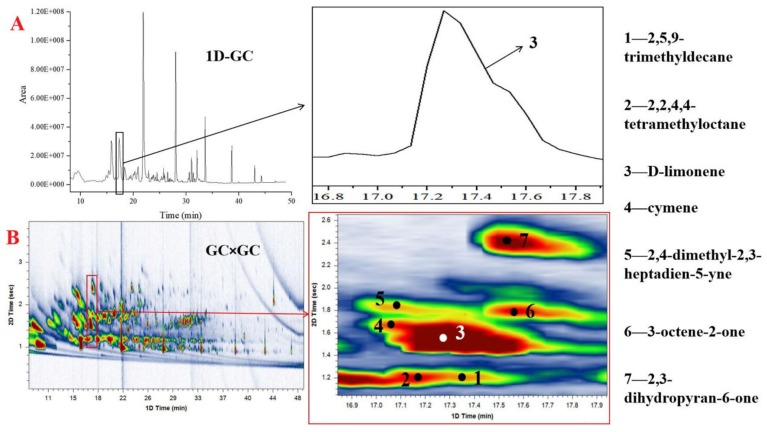
Comparison of an expanded region of the 1D GC (**A**) and the same region from GC × GC (**B**) chromatograms of volatile components from a quality control sample. Compounds identification: (1) 2,5,9-trimethyldecane; (2) 2,2,4,4-tetramethyloctane; (3) d-limonene; (4) o-cymene; (5) 2,4-dimethyl-2,3-heptadien-5-yne; (6) 3-octene-2-one; (7) 2,3-dihydropyran-6-one.

Using the technique of HS-SPME combined with GC×GC-QTOFMS, unknown analytes can be identified and quantified in one GC injection. To compare the techniques of GC-MS and GC×GC-MS, a quality control sample (mix of each collected sample) was analyzed by both techniques under the same chromatographic conditions as described by Xiang et al. [19]. The result shows that the number of detected peaks as well as the chromatographic response in the GC×GC-MS chromatogram significantly increase compared to that of the GC-MS (Figure 1). With the high chromatographic resolution, many overlapped peaks by 1-DGC were resolved by GC×GC. In the quality control sample, the higher resolving power of GC×GC is visibly demonstrated by the constituent D-limonene (peak 3), which appears as a single overlapped peak from 1D-GC (Figure 1A) and can be separated into seven individual peaks by GC×GC (Figure 1B), namely 2,5,9-trimethyldecane, 2,2,4,4-tetramethyloctane, o-cymene, 2,4-dimethyl-2,3-heptadien-5-yne, 3-octene-2-one, and 2,3-dihydropyran-6-one (peaks 1, 2, 4, 5, 6, 7, respectively); these compounds are separated only in the second dimension. The results revealed that the volatile components of *O. sinensis* and insect host products were complex and required GC×GC for complete characterization; GC×GC-MS has a superior sensitivity and resolution, providing an efficient and convenient approach for studying the volatile compounds of these products. Furthermore, the present method is automated and meets the requirement of the principles of green analytical chemistry, such as solvent-free sampling, small amounts of reagents, hermetic sealing of analytical process, reduced waste, and less time consumption [22,23]. 

### 2.2. Identification of Volatile Components

**Figure 2:**
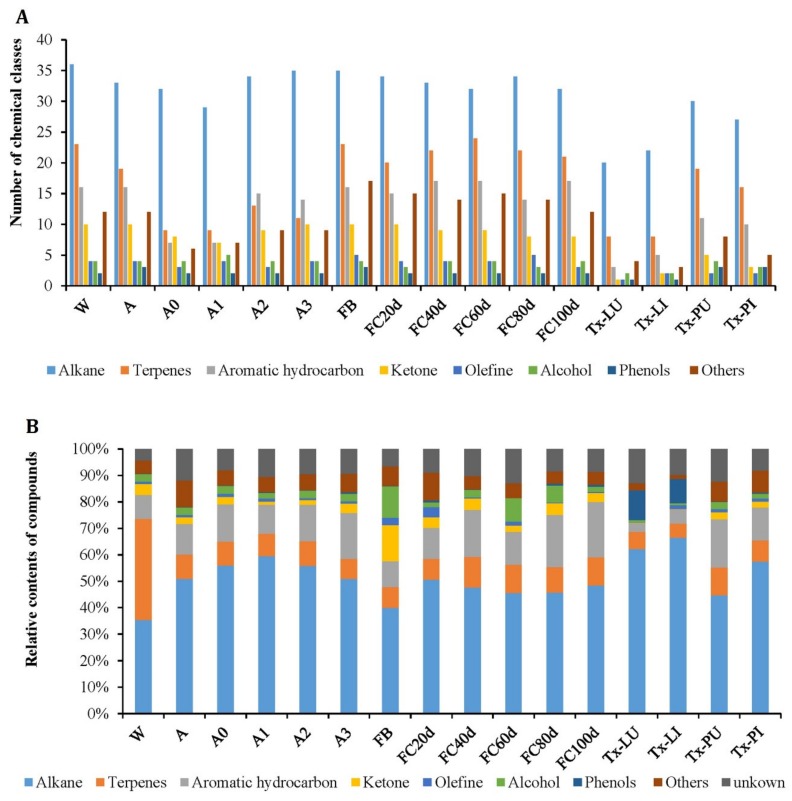
The number (**A**) and relative content (**B**) of volatile compounds of each class in different products. Note: For descriptions of W, A, A0, A1, A2, A3, FB, FC20d, FC40d, FC60d, FC80d, FC100d, Tx-LU, Tx-LI, Tx-PU, and Tx-PI, please refer to Table 1).

Component identification was achieved by matching the QTOFMS spectral with a commercial mass spectral library (NIST 17), with a minimum match factor of 800. The current quantitative method was consistent with similarly reported references [25,26], and the qualitative data of volatile components in different analyzed products (Table 1) with their peak area percentages are presented in Table 2. A total of 120 volatile compounds were detected in all samples with various concentration levels. A total of 107, 101, 71, 70, 89, 89, 113, 103, 105, 107, 102, 99, 40, 45, 82, and 69 compounds (Figure 2A) were identified in the products of W, A, A0, A1, A2, A3, FB, FC20d, FC40d, FC60d, FC80d, FC100d, Tx-LU, Tx-LI, Tx-PU, and Tx-PI (Table 1), accounting for 95.70%, 88.14%, 91.96%, 89.50%, 90.59%, 90.73%, 93.42%, 91.08%, 89.84%, 87.04%, 91.54%, 91.45%, 87.10%, 90.15%, 87.63%, and 91.81% of the total peaks areas, respectively. The identified compounds included 36 alkanes, 25 terpenes, 17 aromatic hydrocarbons, 10 ketones, 5 olefines, 5 alcohols, 3 phenols, and 19 other compounds. The volatile compound amounts showed great variation in different samples and ranged from 40 compounds in Tx-LU to 113 compounds in FB. In general, natural and mature artificial Chinese cordyceps (fungus–insect complexes), fruiting bodies, and fermented products had more volatile compounds than insect larvae, insect pupae and immature artificial Chinese cordyceps. 

Twenty-four volatile compounds were identified in all the 16 samples, including 4-carene (C10), 3-carene (C18), 2,2,4,4-tetramethyloctane (24), 2-propyltoluene (C29), 2,4,6-trimethyldecane (C31), 2,6-dimethyl-6-trifluoroacetoxyoctane (C34), linalool (C41), 2-nonen-1-ol (C42), 6-ethyl-2-methyloctane (C44), (+)-α-terpineol (C61), 2,6-dimethylundecane (C65), 2,8-dimethylundecane (C68), 4-methyldodecane (C73), 4,7-dimethylindan (C75), 2,3-dimethylundecane (C76), 2,4-dimethyldodecane (C77), 2,6,11-trimethyldodecane(C78), 4,6-dimethyldodecane (C84), 2,3,5,8-tetramethyldecane (C86), farnesane (C94), tetradecane (C97), seychellene (C108), 2,6-di-tert-butyl-4-methyl-p-quinol (C109), and hexadecane (C118). The mutual volatile compounds accounted for 40.19%, 41.24%, 51.44%, 48.59%, 50.58%, 41.86%, 38.44%, 48.04%, 48.31%, 47.03%, 44.37%, 48.49%, 64.94%, 68.73%, 49.13%, and 40.87% of the total volatile compounds in W, A, A0, A1, A2, A3, FB, FC20d, FC40d, FC60d, FC80d, FC100d, Tx-LU, Tx-LI, Tx-PU, and Tx-PI, respectively. With the method of simultaneous distillation-extraction (SDE) and GC-MS, 17 and 42 volatile compounds were identified in the mycelia of *O. sinensis* from solid-state media and submerged fermentation, respectively [17]; in Bailing capsule and Zhiling capsule, the commercial fermentation products of *O. sinensis* mycelia, 39 and 56 volatile compounds were identified, respectively [18]. While by the technique of HS-SPME combined with GC×GC-QTOFMS in this study, 99–107 volatile compounds were identified from the fermentation cultures of *O. sinensis* mycelia, indicating the superior sensitivity and resolution of the present method.

### 2.3. Major Compounds in Different Products

The numbers and percentage contents of volatile compounds in samples are of marked differences. Alkanes are the dominant volatile compounds in all samples. Alkane is also the class with the largest number in all samples (Figure 2). The top five compounds in the concentration of each sample are shown in Table 3. 

2,5,6-trimethyldecane (C19) is the most abundant compound in artificial cultivated Chinese cordyceps (A, A0, A1, A2, A3) and insect pupae (Tx-PU and Tx-PI), and it is also the major compound in W, FB, and FC40d, FC80d, and FC100d (Table 3). This compound is so far detected from beneficial plants such as Irish York cabbage [28], stevia *Stevia rebaudiana* leaves [29], an aquatic perennial herb *Limnophila indica* extract [30], plant-based food such as chestnut and jujube honey [31], and from exhaled breath in both children with allergic asthma and control [32]. 5,6-Dihydro-6-pentyl-2*H*-pyran-2-one (massoia lactone) is discovered as the dominant volatile component in a fermented mycelial product of *Paecilomyces hepiali* fungus [18]. 2,5,6-trimethyldecane is the first reported dominant volatile compound in *O. sinensis*-based products in the present study. Moreover, it seemed interesting that uninfected insect pupae also contained high concentrations of this compound. Its characteristics and possible pharmacological functions need further study. 

2,3-dimethylundecane (C76) is another major component presented in 13 samples accounting for >5% of the total peak areas; however, the contents in samples of W, Tx-LU, and Tx-LI were lower, accounting for 2.97%, 1.25%, and 0.91%, respectively (Table 2 and Table 3). This compound was found from the essential oil of a small glabrous, perennial herb *Viola serpens* [33] and from the odors emitted from the dung of free-ranging white rhinos for differentiating sex [34].

A high content of 2,2,4,4-tetramethyloctane (C24) was found in the two larval samples of Tx-LU and Tx-LI, accounting for 56.02% and 61.87% of the total peak areas, although it is not reported from other insects. 2,2,4,4-tetramethyloctane is also the major compound of all the liquid fermentation samples (FC20d, FC40d, FC60d, FC80d, and FC100d). It was reported also in aged vinegar as an aroma compound [35], common wasp *Vespula vulgaris* colonies [36], Manchego and Gouda cheeses [37], *Allium macrostemon* flowers and aerial parts [38], the seeds and leaves of *Synsepalum dulcificum* [39], green teas [40], dry-cured meat products [41], and the stem of Guanyin tea [42]. It appears that this volatile mainly acts as an aroma compound from the plants and foods.

2,4-di-tert-butyl-6-methylphenol (C116) is the second principal component in the two larval samples of Tx-LU and Tx-LI, accounting for 11.27% and 9.14% of the total peak areas, but it accounts for little or no proportion in other samples. This volatile is detected from the essential oil in eaglewood [43] and entomopathogenic *Metarhizium anisopliae* fungus cultures [44].

The most abundant volatile compound was butylated hydroxytoluene, and the major classes compounds were phenols, acids, and alkanes in the mycelia of *O. sinensis* cultured by solid-state media and submerged fermentation [17]. 5,6-Dihydro-6-pentyl-2*H*-pyran-2-one (massoia lactone) was the dominant component in Jinshuibao capsule (*Paecilomyces hepiali*) volatiles, and fatty acids including palmitic acid (C16:0) and linoleic acid (C18:2) were also found to be major volatile compositions in the commercial fermentation products of Bailing capsule (*O. sinensis*), Zhiling capsule (*Mortierella* SP), Ningxinbao capsule (*Cephalosporium sinensis*), and Xinganbao capsule (*Gliocladium roseum*) [18]. In the present study, volatile compounds of alkanes are the most abundant all products, although there are differences among the volatile compound profiles of *O. sinensis* fungus, *Thitarodes* hosts of *O. sinensis*, and the natural and aritificial-producing Chinese cordyceps, even between the natural and artificial-producing Chinese cordyceps. It appeared that *O. sinensis*-based products from different culture conditions exhibit quite different metabolites.

The fermented products of *O. sinensis* mycelia are claimed to be used as sustainable substitutes for natural Chinese cordyceps [45]. However, from the view of the differences in volatile compounds, it seems that the fermented products are not the same as the natural and artificial Chinese cordyceps.

### 2.4. Multivariate PLS-DA Analysis

**Figure 3:**
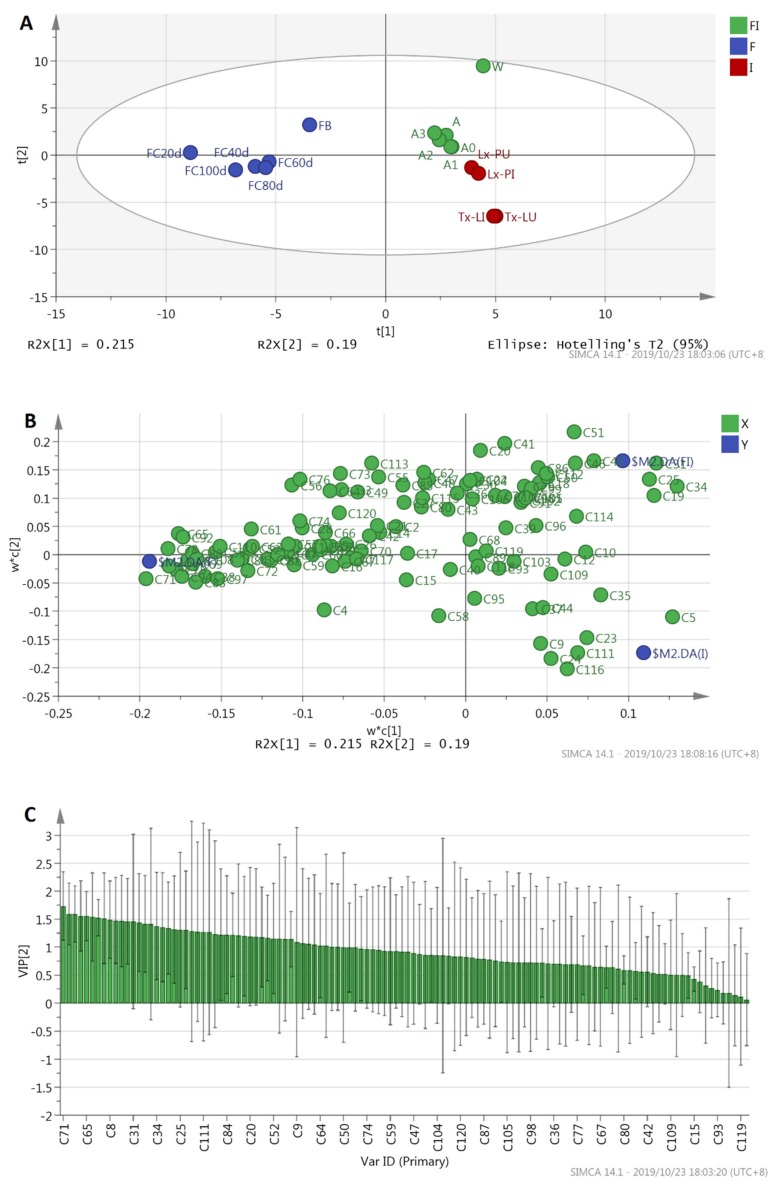
Partial least squares-discriminant analysis (PLS-DA) analysis of 16 products. (**A**): Scores plot, (**B**): variable importance projection (VIP) score plot, (**C**): loadings plot. F: Fungus of *Ophiocordyceps sinensis*; I: Larvae or pupae of *Thitarodes xiaojinensis*; FI: Fungus–insect complexes.

PLS-DA was performed to evaluate the variations among the volatile compound profiles obtained from GC×GC-QTOFMS data for different products. The PLS-DA scores plot shows clear classification of the three groups: fruiting bodies and fermented cultures of *O. sinenis* fungus, *T. xiaojinensis* insects, and insect–fungus complexes (Figure 3A). PLS (Partial least square) component 1 (PLS 1) and PLS component 2 (PLS 2) explained 21.5% and 19.0% of the variance, respectively, and hence together, they explained 40.05% of the total variance. The parameters of the cross-validation modeling for the fifth PLS component were R^2^X = 0.73, R^2^Y = 0.992, and Q^2^Y = 0.896, showing high levels of an explained variance and predictability. A permutation test involving 200 iterations was also conducted to validate the model, which yielded R^2^ = 0.770 and Q^2^ = −0.480.

To explain the relationships between variables and products, loading scatter plots were performed (Figure 3B). As shown in the loadings plot PLS-DA model (Figure 3B), X-variables situated in the vicinity of the dummy Y-variables had the highest discriminatory power among the groups and had higher VIP (variable importance projection) values, thus contributing more to the differences of different groups. The VIP values of each compound were calculated (Figure 3C). The compounds with larger VIP values represent higher contributions to the discrimination of different groups. In the study, volatile components with VIP values > 1 and *p* < 0.05 were considered as representative differential compounds. A total of 28 differential volatile compounds were identified, although there were 48 volatile compounds with VIP values > 1. It showed that the majority of variables gave a not significant contribution to the model. The 28 differential volatile compounds included thieno [2,3-c] pyridine (C71), 2,6,11-trimethyldodecane (C78), 2,3,4-trimethyldodecane (C79), 4,7-dimethylindan (C75), 2,6-dimethylundecane (C65), farnesane (C94), 2,3-dimethyldodecane (C92), [but-2-en-2-yl] benzene (C51), 2-methylcyclopentanone (C8), 2-undecanone (C83), 2,8-dimethylundecane (C69), α-methyl-1*H*-indene-1-methanol acetate (C88), 2,6-dimethylheptadecane (C108), 1,2-dimethoxyethylbenzene (C38), 2,6-dimethyl-6-trifluoroacetoxyoctane (C34), modephene (C97), 4’-methylpropiophenone (C53), 2,6-di-tert-butyl-4-methyl-p-quinol (C110), o-xylene (C5), 3-methyl-undecane (C56), tridecane (C84), 2,5,6-trimethyldecane (C19), sabinene (C13), dodecane (C61), β-sesquiphellandrene (C113), 2,3-dimethyldecane (C52), 3,4-dimethylcumene (C63), and 4,7-dimethylundecane (C64). Among them, the first eight volatiles including thieno [2,3-c] pyridine (C71), 2,6,11-trimethyldodecane (C78), 2,3,4-trimethyldodecane (C79), 4,7-dimethylindan (C75), 2,6-dimethylundecane (C65), farnesane (C94), 2,3-dimethyldodecane (C92), and [but-2-en-2-yl] benzene (C51) showed higher discriminatory potential with VIP values greater than 1.5.

## 3. Materials and Methods

### 3.1. Chemicals

All solvents used were chromatographic grade. Phenylethyl acetate (internal standard) with purity greater than 99.0% was purchased from Sigma-Aldrich-Fluka (Buchs, Switzerland). The internal standard with a concentration of 22.9 µg/mL was prepared in acetonitrile. A standard series of n-alkanes (C8–C25) were provided by Dr. Ehrensorfer (Augsburg, Germany). Methanol (chromatographic grade purity) and acetonitrile (chromatographic grade purity) were purchased from Merck (LiChrosolv, Germany). All chemicals were stored at 4 °C until use. The SPME holder for manual sampling and fibers of 65 µm divinylbenzene/carboxen/polydimethylsiloxane (DVB/CAR/PDMS, 1 cm of length) were purchased from Supelco (Aldrich, Bellefonte, PA, USA).

### 3.2. Samples

The samples for GC-MS analysis are listed in Table 1. Natural Chinese cordyceps were collected from Kangding, Sichuan Province, China. The KD1223 strain of *O. sinensis* fungus isolated from the fruiting bodies of natural Chinese cordyceps was cultured in a 100 rpm shaker with potato dextrose liquid medium supplemented with 10% peptone (PPD) at 13 °C. The fungus was identified by molecular method using the internal transcribed spacer (ITS; ITS1-5.8S-ITS2) of nuclear ribosomal DNA amplification as described before. The identified *O. sinensis* strain was preserved at −80 °C at the Guangdong Institute of Applied Biological Resources, Guangzhou, China.

Artificial cultivation of fruiting bodies on rice media [6] or whole Chinese cordyceps by challenging *T. xiaojinensis* larvae with *O. sinensis* fungus [7,8,11] were established in low altitude Guangzhou, with mimicking environmental conditions. The insect species was identified using a molecular method by the amplification of the Cytochrome b sequences with the primers CB1 (TATGTACTACCATGAGGACAAATATC) and CB2 (ATTACACCTCCTAATTTATTAGGAAT) [46], as described previously [47,48]. The mummified cadavers with mycelia but without fruiting body (before stroma development), and the insect larvae and pupae with or without the injected blastospores (9 months for larvae or 9 months for pupae) were also used for the analysis. The existence of blastospores in the live larvae and pupae was confirmed by hemolymph microscopic examination.

A total of 50 individuals of each sample (for natural and artificial Chinese cordyceps, mummified cadavers, live larvae and pupae with or without blastospores), 30 g of fresh artificial fruiting bodies, and three flasks of fermentation cultures (150 mL/flask) were sampled. Samples were frozen at −80 °C overnight and lyophilized for 48–72 h by vacuum-freeze dryer (Alpha 1-2 LD plus, Marin Christ Gefriertrocknungsanlagen, Osterode, Germany) to consistent weight. The dried samples were grinded at 1000 rpm for 3 min by a multifunctional high-throughput tissue ball mill (GT100, Beijing Grinder Instrument Co., Ltd., Beijing, China) and stored at −80 °C. A quality control (QC) sample was prepared by mixing each collected sample in equal quantities and used for analytical method establishment and methodology examination.

### 3.3. GC×GC-QTOFMS Analysis for Volatile Components

The analysis of volatile composition and analytical method validation were referenced by the method of previous reports [19,21,24]. The volatile constituents of *O. sinensis* and host insects were analyzed by comprehensive two-dimensional gas chromatography (7890B-SSM1800, Agilent Technologies, Santa Clara, CA, USA and J&X Technologies, Shanghai, China) coupled with a high-resolution quadrupole time-of-flight mass spectrometry (QTOFMS) (7250, Agilent Technologies). First, 100 mg of samples were accurately weighed into a 20 mL vial, and then the SPME fiber that was equilibrated at 270 °C for 30 min in an autosampler (PAL RSI 120, CTC Technologies, Alexandria, VA, USA) was exposed to the headspace of the bottle for 20 min at 60 °C. Then, the SPME fiber was introduced into the GC splitless injector and kept there for 3.0 min to allow thermal desorption of the analytes. All samples were conducted in triplicate to check the repeatability and reliability of the method development. Reproducibility is expressed as the relative standard deviation (RSD). To compare the techniques of GC-MS and GC×GC-MS, a quality control sample (mix of each collected sample) was analyzed by both techniques under the same chromatographic conditions. The analytical system was equipped with simultaneous 1DGC and GC×GC in one instrument, which can conduct both techniques at the same time without any change of columns. The samples were introduced by a splitless injector (SSL) system equipped with an autosampler. Peak separation was performed on a weak-polar column HP-5 MS (5% phenyl-95% dimethylpolysiloxane, 30 m × 250 μm, 0.25 μm) in the first dimension and a more polar column DB-17 MS (50% phenyl-50% dimethylpolysiloxane, 1.2 m × 180 μm, 0.18 μm) in the second dimension (both from Agilent Technologies, USA).

The 1DGC and GC×GC conditions were the same. The GC injector was kept at 250 °C in splitless mode. The carrier gas was helium with a flow rate of 1.0 mL/min for the first dimensional column. The initial oven temperature was 50 °C; it was held for 3 min and then ramped at a rate of 4 °C/min to 230 °C and held for 1 min. For the GC×GC system, the carrier gas was helium with a flow rate of 3.14 mL/min for second dimensional column, and the cold zone temperature of modulator was set at −50 °C. The temperatures of the entry hot zone and exit hot zone were +30 and +120 °C relative to oven temperatures, respectively, with a cap temperature of 320 °C for both hot zones. The modulation period was 4 s.

The MS transfer line temperature was kept at 280 °C, and the ion source temperature was kept at 200 °C. Electron impact ionization was 70 eV. Data were collected as a mass range of 50–500 *m*/*z* at a sampling rate of 50 scan/s, and a solvent delay of 8 min was used.

### 3.4. Data Analysis

Qualitative and semi-quantitative methods primarily were referenced with similar reports [26,27]. Compound identification was based on mass spectra comparison with NIST 17 library (NIST/EPA/NIH 2017) with the minimum requirements of match factor above 800. Further confirmation was carried out using one-dimensional retention index (RI) and accurate mass, as described in many previous studies [49]. In order to compare the reference RI values with experimental RI values obtained in this work, a standard mixture of n-alkanes (C8–C25) was injected (0.5 μL) in the GC×GC-QTOFMS system under the same conditions used for the samples. The semi-quantitative method was performed based on peak area normalization. The 1-DGC data were processed using Agilent Mass Hunter Qualitative Analysis Navigator B.08.00. The GC×GC data were analyzed by a dedicated GC×GC data processing software Canvas (V1.4.0, J & X Technologies).

To visualize the clustering among categories and identify the differentially changed components responsible for the separation, supervised partial least squares discriminant analysis (PLS-DA) and variable importance in projection (VIP) score were carried out using SIMCA 14.1 software (Umetrics, Umea, Sweden). A data set consisting of a 16 × 120 matrix was conducted by PLS-DA. The rows represent the samples analyzed and the columns represent the relative contents of the volatile metabolites determined by GC×GC-QTOFMS. All variables were scaled with unit variance (UV) prior to PLS-DA. To gain the chemical markers for discrimination of the three groups in the PLS-DA model, VIP values were calculated and inspected for identified volatile compounds. Generally, VIP values > 1 and *p* < 0.05 are considered as significant contributors to the model [40,50,51]. In this study, seven-fold cross-validation and 200 response permutation testing (RPT) methods were used to investigate the quality of the model.

## 4. Conclusions

This study presents the volatile metabolite profiles by HS-SPME-GC×GC-QTOFMS from *O. sinensis* fungus and insect host-based products. A total of 120 volatile compounds including 36 alkanes, 25 terpenes, 17 aromatic hydrocarbons, 10 ketones, 5 olefines, 5 alcohols, 3 phenols, and 19 other compounds were identified. There are great differences in the volatile compounds among the three categories of *O. sinensis* fungus, *Thitarodes* hosts of *O. sinensis*, and the Chinese cordyceps. In general, natural and mature artificial Chinese cordyceps (fungus–insect complexes), fruiting bodies, and fermented products had more volatile compounds than insect larvae, insect pupae, and immature artificial Chinese cordyceps. Twenty-four volatile compounds were identified in all the 16 samples, including 4-carene (C10), 3-carene (C18), 2,2,4,4-tetramethyloctane (24), 2-propyltoluene (C29), 2,4,6-trimethyldecane (C31), 2,6-dimethyl-6-trifluoroacetoxyoctane (C34), linalool (C41), 2-nonen-1-ol (C42), 6-ethyl-2-methyloctane (C44), (+)-α-terpineol (C61), 2,6-dimethylundecane (C65), 2,8-dimethylundecane (C68), 4-methyldodecane (C73), 4,7-dimethylindan (C75), 2,3-dimethylundecane (C76), 2,4-dimethyldodecane (C77), 2,6,11-trimethyldodecane(C78), 4,6-dimethyldodecane (C84), 2,3,5,8-tetramethyldecane (C86), farnesane (C94), tetradecane (C97), seychellene (C108), 2,6-di-tert-butyl-4-methyl-p-quinol (C109), and hexadecane (C118). Alkanes are the dominant volatile compounds in all products. 2,5,6-trimethyldecane and 2,6,7-trimethyldecane are the major volatile compounds in all products except the larval ones, while 2,2,4,4-tetramethyloctane dominates in the larval products. From the view of the differences in volatile compounds, it seems that the fermented products are not the same as the natural and artificial Chinese cordyceps. Based on the volatile compounds, three classes (*O. sinensis* fungus, *Thitarodes* insect, and fungus–insect complexe) were confirmed by partial least squares-discriminant analysis (PLS-DA). Thieno [2,3-c] pyridine, 2,6,11-trimethyldodecane, 2,3,4-trimethyldodecane, 4,7-dimethylindan, 2,6-dimethylundecane, farnesane, 2,3-dimethyldodecane, and [but-2-en-2-yl] benzene are potential discriminatory compounds. The present results suggested that HS-SPME-GC×GC-QTOFMS combined with multivariate data analysis is an ideal method for analyzing and distinguishing different *O. sinensis* and insect hosts-based products. The information provided in this study is of importance for the further identification of bioactive components and for proposals of possible mechanisms to obtain those bioactive compounds in a different form than the traditional fungus-insect interaction.

## Figures and Tables

**Table 1:** Information.

Sample Code	Sample Description
W	Natural Chinese cordyceps from Kangding, Sichuan, China
A	Artificial cultured Chinese cordyceps by Liyuan Co., Ltd., Guangzhou
A0	The mummified *Thitarodes* larvae before stroma development
A1	Artificial cultivated Chinese cordyceps with fruiting bodies (lengths about 1 cm)
A2	Artificial cultivated Chinese cordyceps with fruiting bodies (lengths about 2–3 cm)
A3	Artificial cultivated Chinese cordyceps with fruiting bodies (lengths about 4–5 cm)
FB	Fruiting bodies of *Ophiocordyceps sinensis* on rice–wheat medium
FC20d	Fermented culture in PM medium (200 g potato extract, 20 g maltose, 10 g peptone, 1.5 g KH_2_PO_4_, 0.5 g MgSO_4_, 20 mg vitamin B1, and 1000 mL distilled water) [11] supplemented with 0.5% *Galleria mellonella* larvae for 20 days, at 11 ± 2 °C
FC40d	Fermented culture of *Ophiocordyceps sinensis* in PM medium [11] supplemented with 0.5% *Galleria mellonella* larvae for 40 days, at 11 ± 2 °C
FC60d	Fermented culture of *Ophiocordyceps sinensis* in PM medium [11] supplemented with 0.5% *Galleria mellonella* larvae for 60 days, at 11 ± 2 °C
FC80d	Fermented culture of *Ophiocordyceps sinensis* in PM medium [11] supplemented with 0.5% *Galleria mellonella* larvae for 80 days, at 11 ± 2 °C
FC100d	Fermented culture of *Ophiocordyceps sinensis* in PM medium [11] supplemented with 0.5% *Galleria mellonella* larvae for 100 days, at 11 ± 2 °C
Tx-LU	Uninfected L5-L6 instar larvae of *Thitarodes xiaojinensisi*
Tx-LI	Infected L5-L6 instar larvae of *Thitarodes xiaojinensisi* with blastospores of *Ophiocordyceps sinensis* fungus
Tx-PU	Uninfected pupae of *Thitarodes xiaojinensisi*
Tx-PI	Infected pupae of *Thitarodes xiaojinensisi* with blastospores of *Ophiocordyceps sinensis* fungus

**Table 2:** Volatile components identified in different products.

NO.	Compound	Class	Retention Time (min)	Formula	RI	Peak Area Percentage (%)															
						W	A	A0	A1	A2	A3	FB	FC20d	FC40d	FC60d	FC80d	FC100d	Tx-LU	Tx-LI	Tx-PU	Tx-PI
C1	3-Prop-2-enylidenecyclobutene	Olefine	8.50	C_7_H_8_	804	0.00	0.52	0.57	1.04	0.53	0.35	0.53	3.84	0.32	1.39	0.13	0.43	0.14	1.22	0.00	0.42
C2	2-Methyl cyclopentanol	Alcohol	8.90	C_6_H1_2_O	815	1.00	2.14	2.20	1.21	1.82	1.83	10.80	0.00	1.16	1.21	0.00	0.54	0.41	0.57	0.82	0.91
C3	Methyl carbamate	Others	9.23	C_2_H_5_NO_2_	824	0.00	0.00	0.00	0.02	0.00	0.00	0.00	0.00	0.00	0.00	0.00	0.00	0.03	0.00	0.00	0.00
C4	4-Methyl pyrimidine	Others	9.83	C_5_H_6_N_2_	842	0.00	0.00	0.00	0.00	0.00	0.00	0.04	0.00	0.15	0.25	0.00	0.07	0.00	0.00	0.17	0.00
C5	o-Xylene	Aromatic hydrocarbon	11.23	C_8_H_10_	881	2.15	2.65	1.57	2.05	1.51	2.86	3.68	0.00	1.04	0.47	0.00	0.35	1.57	3.72	3.93	5.09
C6	2,2-Dimethoxy ethanol	Alcohol	11.57	C_4_H_10_O_3_	890	0.00	0.00	0.00	0.02	0.00	0.00	0.00	0.00	0.00	0.00	0.00	0.00	0.00	0.00	0.00	0.00
C7	N-methoxy formamide	Others	12.03	C_2_H_5_NO_2_	903	0.00	0.00	0.00	0.01	0.00	0.00	0.00	0.00	0.01	0.00	0.01	0.00	0.00	0.00	0.00	0.00
C8	2-Methyl cyclopentanone	Ketone	12.03	C_6_H_10_O	904	0.07	0.04	0.14	0.02	0.32	0.11	0.05	0.65	1.00	0.95	0.83	0.74	0.00	0.00	0.18	0.00
C9	4,6-Dimethyl pyrimidine,	Others	12.97	C_6_H_8_N_2_	928	0.05	0.04	0.00	0.00	0.00	0.00	0.18	0.00	0.00	0.00	0.00	0.00	0.00	0.00	0.75	0.19
C10	4-Carene	Terpenes	13.57	C_10_H_16_	943	4.52	0.02	0.14	0.08	0.13	0.04	0.03	0.04	0.15	0.08	0.06	0.05	1.13	1.09	0.61	0.85
C11	(2,5-Dimethyloxan-2-yl) methanol	Alcohol	14.83	C_8_H_16_O_2_	976	0.50	0.53	0.25	0.28	0.27	0.18	0.57	0.44	0.72	0.87	0.40	0.47	0.00	0.00	0.90	0.16
C12	1-Phenyl-1,2-propanedione	Ketone	14.77	C_9_H_8_O_2_	975	0.94	0.76	1.27	0.13	0.10	1.55	1.52	0.23	0.29	0.34	0.01	0.20	0.00	0.00	1.71	1.85
C13	Sabinene	Terpenes	15.03	C_10_H_16_	981	0.00	0.02	0.08	0.09	0.02	0.08	0.02	0.56	0.63	1.10	0.02	0.36	0.00	0.00	0.00	0.00
C14	6-Ethenyl-2,2,6-trimethyloxan-3-one	Ketone	15.30	C_10_H_16_O_2_	988	0.45	0.51	0.46	0.18	0.34	0.76	5.29	0.12	0.00	0.00	0.00	0.00	0.00	0.00	0.00	0.00
C15	(4-Hydroxyphenyl) phosphonic acid	Others	15.70	C_6_H_7_O_4_P	999	0.49	0.32	0.38	0.03	0.00	0.24	0.03	4.06	0.00	0.19	0.00	0.00	1.04	0.93	0.00	0.00
C16	(−)-β-Pinene	Terpenes	15.70	C_10_H_16_	998	0.00	0.02	0.00	0.00	0.00	0.00	0.82	0.17	0.01	0.01	0.00	0.00	0.00	0.00	0.03	0.00
C17	1,2,3-Trimethyl benzene	Aromatic hydrocarbon	15.90	C_9_H_12_	1004	0.27	0.36	0.16	0.09	0.09	0.24	0.11	0.22	0.39	0.28	0.29	0.25	0.00	0.05	0.04	0.67
C18	3-Carene	Terpenes	16.50	C_10_H_16_	1019	4.78	1.19	1.11	1.31	1.05	0.60	1.17	0.80	0.75	1.43	0.21	0.50	0.29	0.17	1.60	1.14
C19	2,5,6-Trimethyl decane	Alkane	16.57	C_13_H_28_	1020	8.24	21.12	13.94	20.95	17.49	17.56	8.18	0.00	9.40	0.00	7.63	0.00	1.29	1.09	14.60	29.16
C20	2,6,7-Trimethyl decane	Alkane	16.83	C_13_H_28_	1027	2.91	2.12	10.77	8.39	9.75	6.41	3.03	5.63	0.00	9.35	0.00	7.61	0.39	0.29	3.69	3.82
C21	o-Cymene	Aromatic hydrocarbon	17.10	C_10_H_14_	1034	0.19	0.32	1.72	0.00	0.10	7.31	1.41	0.12	0.16	0.21	7.69	5.17	0.99	0.61	0.04	0.21
C22	2,4-Dimethyl-2,3-heptadien-5-yne	Olefine	17.10	C_9_H_12_	1034	0.49	0.08	0.00	0.02	0.05	0.02	0.12	0.03	0.03	0.03	0.10	0.00	0.00	0.00	0.00	0.00
C23	D-Limonene	Terpenes	17.17	C_12_H_26_	1036	0.32	2.38	0.07	0.00	0.08	0.00	0.00	0.21	0.38	0.36	0.31	0.30	3.22	2.55	0.12	0.71
C24	2,2,4,4-Tetramethyl octane	Alkane	17.23	C_10_H_16_	1037	11.42	1.19	3.24	6.32	2.61	4.13	2.55	6.73	9.70	8.54	7.56	5.38	56.02	61.87	0.68	1.50
C25	2,5,9-Trimethyl decane	Alkane	17.37	C_13_H_28_	1041	0.76	4.31	1.17	1.58	1.42	1.13	0.70	0.00	0.00	0.00	0.08	0.00	0.33	0.00	1.83	1.37
C26	2,3-Dihydro pyran-6-one	Ketone	17.57	C_5_H_6_O_2_	1046	0.25	0.15	0.04	0.05	0.09	0.25	2.36	0.17	0.18	0.09	0.02	0.07	0.00	0.00	0.00	0.00
C27	3-Octene-2-one	Ketone	17.57	C_8_H_14_O	1046	1.39	0.30	0.34	0.19	0.19	0.33	2.50	0.24	0.01	0.19	0.00	0.00	0.00	0.00	0.00	0.00
C28	2,7,10-Trimethyl dodecane	Alkane	18.23	C_15_H_32_	1063	0.15	0.04	0.00	0.00	0.07	0.11	0.51	0.10	0.04	0.11	0.10	0.07	0.00	0.00	0.00	0.00
C29	2-Propyl toluene	Aromatic hydrocarbon	18.10	C_10_H_14_	1060	2.42	6.47	9.82	8.11	10.91	5.65	2.94	4.54	5.25	7.38	4.70	4.78	0.63	0.85	11.44	5.72
C30	2-Ethyl-1,4-dimethyl benzene	Aromatic hydrocarbon	18.37	C_10_H_14_	1066	0.47	0.06	0.00	0.00	0.01	0.06	0.09	0.01	0.01	0.09	0.06	0.06	0.00	0.00	0.00	0.00
C31	2,4,6-Trimethyl decane	Alkane	18.43	C_13_H_28_	1068	2.09	4.14	8.78	5.02	5.90	3.98	2.79	0.45	0.63	0.58	0.54	0.51	0.25	0.30	5.91	3.64
C32	Phenylglyoxyl monohydrate	Others	18.77	C_8_H_8_O_3_	1077	0.18	0.11	0.03	0.00	0.07	0.14	0.52	0.46	0.16	0.02	0.16	0.14	0.00	0.00	0.24	0.00
C33	3,5-Octadien-2-one	Ketone	18.83	C_8_H_12_O	1078	0.16	0.06	0.00	0.00	0.00	0.04	0.11	0.01	0.09	0.05	0.09	0.03	0.00	0.00	0.00	0.00
C34	2,6-Dimethyl-6-trifluoroacetoxyoctane	Others	19.10	C_12_H_21_F_3_O_2_	1085	3.29	8.16	4.93	5.34	5.61	5.30	4.23	0.73	0.75	0.79	0.63	0.56	1.48	0.33	5.71	7.31
C35	1,3-Dimethyl-3-ethyl benzene	Aromatic hydrocarbon	19.17	C_10_H_14_	1087	0.39	0.02	0.00	0.00	0.04	0.10	0.02	0.03	0.05	0.04	0.03	0.02	0.00	0.13	0.28	0.24
C36	Piperityl acetate	Others	19.57	C_12_H_20_O_2_	1097	0.50	0.10	0.00	0.00	0.04	0.10	0.31	0.03	0.04	0.07	0.02	0.04	0.00	0.00	0.09	0.00
C37	1,2,4,5-Tetramethyl benzene	Aromatic hydrocarbon	19.43	C_10_H_14_	1093	0.30	0.05	0.00	0.00	0.01	0.07	0.21	0.02	0.01	0.03	0.04	0.01	0.00	0.00	1.05	0.00
C38	1,2-Dimethoxyethyl benzene	Aromatic hydrocarbon	19.43	C_10_H_14_O_2_	1094	0.00	0.00	0.00	0.00	0.03	0.02	0.00	6.28	8.33	1.12	6.72	7.70	0.00	0.00	0.29	0.03
C39	Dodecane,2,6,11-trimethyl	Alkane	19.50	C_15_H_32_	1095	0.49	0.07	0.00	0.00	0.00	0.04	0.01	0.02	0.10	0.10	0.04	0.06	0.07	0.02	0.16	0.00
C40	4,5-Dimethylnonane	Alkane	19.97	C_11_H_24_	1107	0.46	0.04	0.59	0.25	0.57	0.81	0.03	1.64	0.70	0.00	0.02	0.77	0.36	0.24	0.74	0.74
C41	Linalool	Terpenes	19.90	C_10_H_18_O	1105	1.60	3.78	4.48	3.76	4.60	3.44	2.27	2.34	2.50	3.99	2.12	2.21	0.67	0.70	4.13	2.33
C42	2-Nonen-1-ol	Alcohol	20.03	C_9_H_18_O	1109	1.20	0.02	0.59	0.52	0.57	0.81	0.30	1.03	0.70	0.98	0.73	0.77	0.36	0.24	0.74	0.74
C43	1,2,3,5-Tetramethyl benzene	Aromatic hydrocarbon	20.30	C_10_H_14_	1116	0.23	0.06	0.00	0.00	0.04	0.08	0.14	0.06	0.04	0.05	0.01	0.05	0.00	0.00	0.05	0.08
C44	6-Ethyl-2-methyl octane	Alkane	20.43	C_11_H_24_	1119	0.53	2.20	0.97	0.63	0.73	0.72	0.47	0.96	1.13	0.41	1.16	1.04	0.68	0.75	1.16	3.42
C45	1,5,6,7-Tetramethyl bicyclo[3.2.0]hepta-2,6-diene	Olefine	20.57	C_11_H_16_	1123	0.14	0.11	0.42	0.10	0.21	0.34	0.21	0.03	0.02	0.02	0.02	0.02	0.00	0.00	0.31	0.00
C46	1,2,3,4-Tetramethyl benzene	Aromatic hydrocarbon	20.83	C_10_H_14_	1130	1.29	0.31	0.18	0.26	0.23	0.26	0.28	0.04	0.03	0.04	0.03	0.03	0.00	0.00	0.25	0.16
C47	3,5-Diethyl-1-methyl benzene	Aromatic hydrocarbon	21.43	C_11_H_16_	1146	0.12	0.12	0.00	0.00	0.00	0.00	0.07	0.04	0.03	0.04	0.00	0.01	0.00	0.00	0.00	0.00
C48	4-Methyl indane	Others	21.57	C_10_H_12_	1149	0.10	0.03	0.00	0.00	0.01	0.01	0.03	0.01	0.00	0.01	0.05	0.01	0.00	0.00	0.00	0.00
C49	1,4-Diethyl-2-methyl benzene	Aromatic hydrocarbon	21.70	C_11_H_16_	1153	0.27	0.07	0.00	0.01	0.03	0.06	0.14	0.23	0.01	0.12	0.02	0.06	0.00	0.00	0.00	0.00
C50	1,2,3,4-Tetramethyl fulvene	Others	22.03	C_10_H_14_	1162	0.08	0.16	0.04	0.07	0.04	0.12	0.07	0.10	0.01	0.03	0.04	0.00	0.00	0.00	0.13	0.03
C51	[but-2-en-2-yl]Benzene	Aromatic hydrocarbon	21.97	C_10_H_12_	1160	0.40	0.19	0.07	0.13	0.15	0.16	0.12	0.10	0.01	0.03	0.04	0.02	0.00	0.00	0.13	0.03
C52	2,3-Dimethyl decane	Alkane	22.10	C_12_H_26_	1163	0.12	0.98	0.37	0.22	0.25	0.19	0.18	0.77	0.76	2.27	2.47	0.82	0.00	0.02	0.26	0.34
C53	4’-Methyl propiophenone	Ketone	22.50	C_10_H_12_O	1174	0.23	0.52	0.56	0.51	0.61	0.41	0.42	2.34	2.37	0.53	3.39	2.26	0.00	0.02	0.56	0.24
C54	5-Butan-2-ylnonane	Alkane	22.30	C_13_H_28_	1168	0.14	0.78	0.18	0.18	0.17	0.17	0.19	0.07	2.08	0.00	2.20	0.09	0.00	0.00	0.22	0.20
C55	9-Methyl heptadecane	Alkane	22.50	C_18_H_38_	1174	0.16	0.09	0.02	0.00	0.02	0.02	0.12	0.11	0.04	0.04	0.02	0.00	0.00	0.00	0.00	0.00
C56	3-Methyl undecane	Alkane	22.70	C_12_H_26_	1179	0.09	0.08	0.02	0.00	0.02	0.05	0.12	0.12	0.09	0.04	0.02	0.03	0.00	0.00	0.00	0.00
C57	1-Ethyl-2,4,5-trimethyl benzene	Aromatic hydrocarbon	22.63	C_11_H_16_	1177	0.20	0.77	0.56	0.43	0.55	0.39	0.45	0.00	2.24	2.60	0.07	2.26	0.00	0.00	0.68	0.25
C58	1-Methyl-cyclododecene	Olefine	22.83	C_13_H_24_	1183	0.23	0.17	0.08	0.05	0.00	0.07	1.91	0.02	0.05	0.05	0.04	0.02	0.00	0.22	0.93	0.73
C59	3,7,11-Trimethyl dodecan-1-ol	Alcohol	23.30	C_15_H_32_O	1195	0.11	0.25	0.01	0.01	0.07	0.09	0.10	0.20	0.22	5.81	5.15	0.17	0.00	0.00	0.11	0.00
C60	Dodecane	Alkane	23.57	C_12_H_26_	1202	0.02	0.00	0.00	0.00	0.01	0.05	0.42	0.05	0.04	0.04	0.02	0.03	0.00	0.00	0.00	0.00
C61	(+)-α-Terpineol	Terpenes	23.50	C_10_H_18_O	1200	1.26	1.23	2.87	2.49	3.06	2.47	2.14	2.35	5.94	2.38	5.64	6.01	0.29	0.28	2.82	1.93
C62	3,4-Dimethyl cumene	Aromatic hydrocarbon	23.70	C_11_H_16_	1206	0.18	0.04	0.00	0.00	0.05	0.05	0.11	0.02	0.09	0.01	0.02	0.02	0.00	0.00	0.00	0.00
C63	4,7-Dimethyl undecane	Alkane	23.77	C_13_H_28_	1208	0.25	1.13	0.76	0.55	0.55	0.52	0.64	1.06	1.67	1.06	1.63	1.10	0.00	0.06	0.56	1.29
C64	Cyclodecanol	Others	23.83	C_10_H_20_O	1210	0.12	0.14	0.04	0.08	0.07	0.09	0.15	0.33	0.08	0.02	0.08	0.07	0.00	0.00	0.03	0.04
C65	2,6-Dimethyl undecane	Alkane	24.10	C_13_H_28_	1217	0.61	0.95	1.24	1.06	1.28	1.02	1.47	3.87	2.49	2.32	2.32	3.43	0.08	0.08	1.13	0.46
C66	2,4-Dimethyl acetophenone	Ketone	24.17	C_10_H_12_O	1219	0.06	0.03	0.02	0.00	0.02	0.03	0.06	0.04	0.28	0.02	0.02	0.01	0.00	0.00	0.00	0.00
C67	2-Hydroxycineole	Others	24.23	C_10_H_18_O_2_	1221	0.00	0.01	0.00	0.00	0.01	0.00	0.47	0.01	0.01	0.01	0.01	0.00	0.00	0.00	0.00	0.00
C68	2,8-Dimethyl undecane	Alkane	24.37	C_13_H_28_	1225	0.11	0.12	0.39	0.33	0.40	0.28	0.38	0.28	0.20	0.23	0.21	0.26	0.06	0.04	0.42	0.48
C69	2,6-Dimethyl benzaldehyde	Others	24.43	C_9_H_10_O	1227	0.01	0.00	0.00	0.00	0.05	0.06	0.01	0.42	0.21	0.29	0.20	0.23	0.00	0.00	0.00	0.00
C70	2-Hydroxycineol	Others	24.63	C_10_H_18_O_2_	1232	0.00	0.10	0.00	0.00	0.00	0.00	0.51	0.02	0.00	0.00	0.00	0.00	0.00	0.00	0.00	0.00
C71	Thieno[2,2,3]pyridine	Others	24.83	C_7_H_5_NS	1239	0.00	0.00	0.00	0.00	0.00	0.00	0.07	0.10	0.05	0.06	0.06	0.05	0.00	0.00	0.00	0.00
C72	3-Hydroxy cineole	Others	25.23	C_10_H_18_O_2_	1249	0.00	0.00	0.00	0.00	0.00	0.00	0.30	0.10	0.10	0.06	0.03	0.03	0.00	0.00	0.00	0.00
C73	4-Methyl dodecane	Alkane	25.57	C_13_H_28_	1258	0.81	1.52	1.43	1.74	1.84	1.68	2.44	2.22	1.23	1.39	1.26	1.40	0.18	0.10	1.00	1.89
C74	1,3-Di-tert-butyl benzene	Aromatic hydrocarbon	25.70	C_14_H_22_	1262	0.04	0.01	0.00	0.00	0.01	0.00	0.07	0.01	0.02	0.01	0.00	0.03	0.00	0.00	0.00	0.00
C75	4,7-Dimethyl indan	Others	25.63	C_11_H_14_	1261	0.13	0.89	0.27	0.24	0.28	0.96	0.39	3.83	3.36	3.66	3.34	3.69	0.15	0.12	0.33	0.75
C76	2,3-Dimethyl undecane	Alkane	25.70	C_13_H_28_	1262	2.97	5.75	6.96	6.91	7.49	6.84	7.11	7.59	6.91	7.50	6.77	7.64	1.25	0.91	7.15	5.78
C77	2,4-Dimethyl dodecane	Alkane	25.90	C_14_H_30_	1268	0.22	0.25	0.74	0.63	0.28	0.55	0.95	0.21	0.16	0.18	0.20	0.25	0.14	0.12	0.49	0.37
C78	2,6,11-Trimethyl dodecane	Alkane	26.43	C_15_H_32_	1283	0.49	0.85	1.42	1.19	1.64	1.14	2.27	4.41	4.14	2.16	3.96	4.52	0.19	0.18	1.45	0.57
C79	2,3,4-Trimethyl dodecane	Alkane	26.77	C_13_H_28_	1292	0.10	0.21	0.44	0.32	0.33	0.25	0.48	2.58	1.61	2.21	1.93	2.43	0.00	0.02	0.31	0.25
C80	1,5,6,7-Tetramethylbicyclo[3.2.0]hepta-2,6-diene	Olefine	26.77	C_11_H_16_	1293	0.05	0.00	0.00	0.00	0.00	0.00	0.02	0.00	0.00	0.00	0.01	0.00	0.00	0.00	0.00	0.00
C81	1,7,7-Trimethylbicyclo[2.2.1]heptan-2-yl acetate	Others	26.90	C_12_H_20_O_2_	1296	0.01	0.00	0.00	0.00	0.00	0.00	0.01	0.04	0.09	0.01	0.01	0.01	0.00	0.00	0.00	0.00
C82	2-Undecanone	Ketone	26.97	C_11_H_22_O	1298	0.20	0.09	0.00	0.00	0.06	0.08	1.10	0.13	0.09	0.12	0.09	0.12	0.00	0.00	0.08	0.00
C83	Tridecane	Alkane	27.17	C_13_H_28_	1304	0.17	0.00	0.11	0.00	0.14	0.15	0.00	2.78	1.44	2.13	1.30	2.34	0.15	0.06	0.32	0.17
C84	4,6-Dimethyl dodecane	Alkane	27.23	C_14_H_30_	1306	0.12	0.24	0.28	0.41	0.32	0.28	0.63	2.04	0.26	1.61	0.27	1.72	0.20	0.08	0.34	0.13
C85	1-Methyl naphthalene	Aromatic hydrocarbon	27.30	C_11_H_10_	1308	0.07	0.04	0.00	0.00	0.00	0.00	0.04	0.01	0.01	0.01	0.02	0.01	0.00	0.00	0.00	0.00
C86	2,3,5,8-Tetramethyl decane	Alkane	27.43	C_14_H_30_	1312	0.68	1.50	0.94	1.51	1.52	1.48	2.42	0.43	0.31	0.29	0.27	0.38	0.22	0.08	1.20	0.93
C87	α-Methyl-1*H*-indene-1-methanol acetate	Others	27.90	C_13_H_14_O_2_	1326	0.08	0.02	0.00	0.00	0.00	0.00	0.03	0.26	0.01	0.04	0.03	0.01	0.00	0.00	0.00	0.00
C88	2,7,10-Trimethyl dodecane	Alkane	28.37	C_15_H_32_	1339	0.09	0.29	0.17	0.16	0.18	0.17	0.33	1.57	0.61	0.74	0.67	1.01	0.00	0.00	0.19	0.06
C89	Silphiperfol-5-ene	Terpenes	28.37	C_15_H_24_	1339	0.17	0.00	0.00	0.00	0.00	0.00	0.07	0.01	0.03	0.02	0.02	0.01	0.00	0.00	0.12	0.00
C90	4-Ethyl undecane	Alkane	28.97	C_13_H_28_	1357	0.08	0.11	0.06	0.10	0.09	0.09	0.18	0.20	0.00	0.21	0.44	0.68	0.00	0.00	0.13	0.14
C91	Silphinene	Terpenes	29.10	C_15_H_24_	1361	0.45	0.02	0.00	0.00	0.00	0.00	0.03	0.01	0.00	0.01	0.02	0.00	0.00	0.00	0.05	0.00
C92	2,3-Dimethyl dodecane	Alkane	29.30	C_14_H_30_	1367	0.05	0.13	0.12	0.16	0.14	0.18	0.11	0.44	0.37	0.48	0.44	0.61	0.00	0.00	0.07	0.08
C93	α-Longipinene	Terpenes	29.30	C_15_H_24_	1367	0.10	0.02	0.00	0.00	0.00	0.00	0.01	0.01	0.02	0.01	0.02	0.02	0.00	0.00	0.10	0.00
C94	farnesane	Alkane	29.70	C_15_H_32_	1378	0.05	0.08	0.03	0.03	0.05	0.05	0.19	0.21	0.25	0.28	0.41	0.58	0.07	0.04	0.04	0.06
C95	(+)-Cyclosativene	Terpenes	29.83	C_15_H_24_	1382	0.15	0.01	0.00	0.00	0.00	0.00	0.14	0.01	0.02	0.02	0.02	0.02	0.00	0.00	0.10	0.13
C96	Modephene	Terpenes	30.37	C_15_H_24_	1398	1.00	0.02	0.00	0.00	0.00	0.00	0.05	0.02	0.06	0.01	0.03	0.03	0.00	0.00	0.20	0.20
C97	Tetradecane	Alkane	30.50	C_14_H_30_	1402	0.27	0.36	0.44	0.50	0.01	0.02	0.51	1.64	0.68	0.61	0.92	1.77	0.24	0.18	0.38	0.38
C98	α-Isocomene	Terpenes	30.57	C_15_H_24_	1404	2.56	0.02	0.00	0.00	0.00	0.00	0.05	0.00	0.05	0.01	0.01	0.02	0.00	0.00	0.05	0.03
C99	(−)-α-Gurjunene	Terpenes	30.97	C_15_H_24_	1417	6.99	0.02	0.07	0.06	0.12	0.60	0.10	0.07	0.03	0.03	0.03	0.04	0.00	0.00	0.06	0.00
C100	2,6-Dimethyl heptadecane	Alkane	31.23	C_19_H_40_	1425	0.04	0.08	0.08	0.05	0.11	0.08	0.09	0.91	0.02	0.30	0.03	0.92	0.00	0.00	0.09	0.00
C101	β-Maaliene	Terpenes	31.30	C_15_H_24_	1428	3.45	0.03	0.00	0.00	0.01	0.02	0.05	0.03	0.01	0.06	0.01	0.02	0.00	0.00	0.07	0.07
C102	(−)-Aristolene	Terpenes	31.57	C_15_H_24_	1436	1.57	0.00	0.00	0.00	0.01	0.02	0.05	0.02	0.01	0.03	0.03	0.00	0.00	0.00	0.00	0.11
C103	2-Isopropyl-5-methyl-9-methylene[4.4.0]dec-1-ene	Terpenes	31.70	C_15_H_24_	1441	0.29	0.01	0.00	0.00	0.00	0.00	0.05	0.00	0.03	0.03	0.05	0.05	0.00	0.00	0.12	0.12
C104	α-Bergamotene	Terpenes	31.90	C_15_H_24_	1447	0.32	0.02	0.00	0.08	0.01	0.00	0.06	0.01	0.02	0.06	0.00	0.07	0.00	0.00	0.00	0.00
C105	Isoledene	Terpenes	32.03	C_15_H_24_	1451	6.98	0.00	0.00	0.00	0.00	0.00	0.04	0.04	0.03	0.04	0.03	0.02	0.00	0.00	0.12	0.05
C106	Guaia-3,9-diene	Terpenes	32.23	C_15_H_24_	1458	0.25	0.00	0.00	0.00	0.00	0.00	0.03	0.01	0.01	0.01	0.02	0.04	0.00	0.00	0.00	0.00
C107	2,10-Dimethyl heptadecane	Alkane	32.50	C_19_H_40_	1466	0.58	0.00	0.00	0.00	0.00	0.00	0.03	0.06	0.01	0.01	0.00	0.00	0.00	0.00	0.00	0.00
C108	Seychellene	Terpenes	32.43	C_15_H_24_	1464	0.17	0.22	0.21	0.28	0.23	0.24	0.46	1.21	0.77	0.20	0.92	0.89	0.14	0.11	0.20	0.17
C109	2,6-Di-tert-butyl-4-methyl-p-quinol	Ketone	32.97	C_15_H_24_O_2_	1481	0.38	0.04	0.04	0.06	0.01	0.06	0.24	0.02	0.01	0.04	0.04	0.01	0.22	0.12	0.08	0.10
C110	Pentadecane	Alkane	33.30	C_15_H_32_	1491	0.05	0.11	0.12	0.19	0.16	0.16	0.23	1.18	0.48	0.17	0.57	0.59	0.00	0.00	0.11	0.00
C111	(+)-Valencene	Terpenes	33.30	C_15_H_24_	1492	0.22	0.00	0.00	0.00	0.00	0.00	0.01	0.00	0.05	0.02	0.02	0.03	0.83	0.42	0.03	0.03
C112	β-Sesquiphellandrene	Terpenes	33.57	C_15_H_24_	1500	0.87	0.09	0.02	0.22	0.07	0.07	0.14	0.01	0.01	0.03	0.01	0.01	0.00	0.00	0.03	0.08
C113	1-Iodo-2-methyl undecane	Alkane	33.90	C_12_H_25_I	1511	0.04	0.04	0.04	0.05	0.16	0.41	0.11	0.15	0.10	0.11	0.12	0.15	0.00	0.00	0.00	0.00
C114	α-Bulnesene	Terpenes	33.97	C_15_H_24_	1514	0.19	0.04	0.00	0.00	0.02	0.06	0.02	0.00	0.00	0.00	0.02	0.03	0.07	0.03	0.00	0.04
C115	2,4-Di-tert-butyl phenol	Phenols	34.17	C_14_H_22_O	1520	0.12	0.04	0.13	0.25	0.16	0.51	0.10	0.75	0.26	0.00	0.00	0.00	0.00	0.00	0.11	0.08
C116	2,4-Di-tert-butyl-6-methyl phenol	Phenols	34.30	C_15_H_24_O	1525	0.00	0.01	0.00	0.00	0.00	0.00	0.08	0.00	0.00	0.02	0.70	0.76	11.27	9.14	0.10	0.21
C117	(+)-Cuparene	Terpenes	34.37	C_15_H_22_	1527	0.06	0.00	0.00	0.00	0.00	0.00	0.00	0.00	0.00	0.80	0.00	0.00	0.00	0.00	0.00	0.00
C118	Hexadecane	Alkane	36.70	C_16_H_34_	1605	0.06	0.09	0.11	0.12	0.06	0.11	0.08	0.11	0.06	0.02	0.12	0.15	0.02	0.02	0.12	0.24
C119	2,6-Di-tert-butyl-4-sec-butyl phenol	Phenols	37.97	C_18_H_30_O	1649	0.01	0.03	0.03	0.03	0.02	0.03	0.01	0.02	0.02	0.02	0.02	0.02	0.00	0.00	0.03	0.06
C120	Pristane	Alkane	39.70	C_19_H_40_	1710	0.02	0.02	0.03	0.04	0.03	0.05	0.05	0.03	0.04	0.04	0.04	0.03	0.00	0.00	0.02	0.06

Note: For descriptions of W, A, A-0, A-1, A-2, A-3, FB, FC20d, FC40d, FC60d, FC80d, FC100d, Tx-LU, Tx-LI, Tx-PU, and Tx-PI, please refer to Table 1.

**Table 3:** Top five compounds in concentration from different products.

Sample	Top Five Compounds									
	1		2		3		4		5	
	Compound No.	Peak Area Percentage (%)	Compound No.	Peak Area Percentage (%)	Compound No.	Peak Area Percentage (%)	Compound No.	Peak Area Percentage (%)	Compound No.	Peak Area Percentage (%)
W	C24	11.42	C19	8.24	C99	6.99	C105	6.98	C17	4.78
A	C19	21.12	C34	8.16	C29	6.47	C76	5.75	C25	4.31
A0	C19	13.94	C20	10.77	C29	9.82	C31	8.78	C76	6.96
A1	C19	20.95	C20	8.39	C29	8.11	C76	6.91	C24	6.32
A2	C19	17.49	C29	10.91	C20	9.75	C76	7.49	C31	5.90
A3	C19	17.56	C21	7.31	C76	6.84	C20	6.41	C29	5.65
FB	C2	10.80	C19	8.18	C76	7.11	C14	5.29	C34	4.23
FC20d	C76	7.59	C24	6.73	C38	6.28	C20	5.63	C29	4.54
FC40d	C24	9.70	C19	9.40	C38	8.33	C76	6.91	C61	5.94
FC60d	C20	9.35	C24	8.54	C76	7.50	C29	7.38	C59	5.81
FC80d	C21	7.69	C19	7.63	C24	7.56	C76	6.77	C38	6.72
FC100d	C38	7.70	C76	7.64	C20	7.61	C61	6.01	C24	5.38
Tx-LU	C24	56.02	C116	11.27	C23	3.22	C5	1.57	C34	1.48
Tx-LI	C24	61.87	C116	9.14	C5	3.72	C23	2.55	C1	1.22
Tx-PU	C19	14.60	C29	11.44	C76	7.15	C31	5.91	C34	5.71
Tx-PI	C19	29.16	C34	7.31	C76	5.78	C29	5.72	C5	5.09

Note: C1: 3-Prop-2-enylidenecyclobutene; C2: 2-methylcyclopentanol; C5: o-xylene; C14: 6-ethenyl-2,2,6-trimethyloxan-3-one; C17: 1,2,3-trimethylbenzene; C19: 2,5,6-trimethyldecane; C20: 2,6,7-trimethyldecane; C21: o-cymene; C23: D-limonene; C24: 2,2,4,4-tetramethyloctane; C25: 2,5,9-trimethyldecane; C29: 2-propyltoluene; C31: 2,4,6-trimethyldecane; C34: 2,6-dimethyl-6-trifluoroacetoxyoctane; C38: 1,2-dimethoxyethylbenzene; C59: 3,7,11-trimethyldodecan-1-ol; C61: (+)-α-terpineol; C76: 2,3-dimethylundecane; C99: (−)-α-gurjunene; C105: isoledene; C116: 2,4-ditert-butyl-6-methyl phenol. The compounds corresponding to the compound numbers are also shown in Table 2.

## References

[1] Li S.P., Yang F.Q., Tsim K.W. (2006). Quality control of *Cordyceps sinensis*, a valued traditional Chinese medicine. J. Pharm. Biomed. Anal..

[2] Zhou X.W., Gong Z.H., Su Y., Lin J., Tang K.X. (2009). *Cordyceps* fungi: Natural products, pharmacological functions and developmental products. J. Pharm. Pharmacol..

[3] Shashidhar M.G., Giridhar P., Sankar K.U., Manohar B. (2013). Bioactive principles from *Cordyceps sinensis*: A potent food supplement–A review. J. Funct. Food.

[4] Yue K., Ye M., Zhou Z.J., Sun W., Lin X. (2013). The genus *Cordyceps*: A chemical and pharmacological review. J. Pharm. Pharmacol..

[5] Li Y., Wang X.L., Jiao L., Jiang Y., Li H., Jiang S.P., Lhosumtseiring N., Fu S.Z., Dong C.H., Zhan Y. (2011). A survey of the geographic distribution of *Ophiocordyceps sinensis*. J. Microbiol..

[6] Cao L., Ye Y., Han R. (2015). Fruiting body production of the medicinal Chinese caterpillar mushroom, *Ophiocordyceps sinensis* (Ascomycetes), in artificial medium. Int. J. Med. Mushrooms.

[7] Cao L., Han R.C. (2014). Artificial Cultivation of Host Insects of *Ophiocordyceps sinensis* in Low Altitude Areas. Chinese Patent.

[8] Tao Z., Cao L., Zhang Y., Ye Y., Han R. (2016). Laboratory rearing of *Thitarodes armoricanus* and *Thitarodes jianchuanensis* (Lepidoptera: Hepialidae), hosts of the Chinese medicinal fungus *Ophiocordyceps sinensis* (Hypocreales: Ophiocordycipitaceae). J. Econ. Entomol..

[9] Yan J.K., Wang W.Q., Wu J.Y. (2014). Recent advances in *Cordyceps sinensis* polysaccharides: Mycelial fermentation, isolation, structure, and bioactivities: A review. J. Funct. Food.

[10] Li X., Liu Q., Li W., Li Q., Qian Z., Liu X., Dong C. (2019). A breakthrough in the artificial cultivation of Chinese cordyceps on a large-scale and its impact on science, the economy, and industry. Crit. Rev. Biotechnol..

[11] Liu G., Han R., Cao L. (2019). Artificial cultivation of the Chinese cordyceps from injected ghost moth larvae. Environ. Entomol..

[12] Yuan J.P., Zhao S.Y., Wang J.H., Kuang H.C., Liu X. (2008). Distribution of nucleosides and nucleobases in edible fungi. J. Agric. Food Chem..

[13] Zhao J., Xie J., Wang L.Y., Li S.P. (2014). Advanced development in chemical analysis of *Cordyceps*. J. Pharm. Biomed. Anal..

[14] Liu Y., Wang J.H., Wang W., Zhang H.Y., Zhang X.L., Han C.C. (2015). The chemical constituents and pharmacological actions of *Cordyceps sinensis*. Evid.-Based Complement. Altern. Med..

[15] Mi J., Han Y., Xu Y., Kou J., Li W.J., Wang J.R., Jiang Z.H. (2018). Deep profiling of immunosuppressive glycosphingolipids and sphingomyelins in wild Cordyceps. J. Agric. Food. Chem..

[16] Yang F.Q., Feng K., Zhao J., Li S.P. (2009). Analysis of sterols and fatty acids in natural and cultured *Cordyceps* by one-step derivatization followed with gas chromatography–mass spectrometry. J. Pharm. Biomed. Anal..

[17] Yu S., Zhang Y., Fan M. (2012). Analysis of volatile compounds of mycelia of *Hirsutella sinensis*, the anamorph of *Ophiocordyceps sinensis*. Appl. Mech. Mater..

[18] Zhang H., Li Y., Mi J., Zhang M., Wang Y., Jiang Z., Hu P. (2017). GC-MS profiling of volatile components in different fermentation products of *Cordyceps sinensis* mycelia. Molecules.

[19] Xiang Z., Chen X., Zhao Z., Xiao X., Guo P., Song H., Yang X., Huang M. (2018). Analysis of volatile components in *Dalbergia cochinchinensis* Pierre by a comprehensive two-dimensional gas chromatography with mass spectrometry method using a solid-state modulator. J. Sep. Sci..

[20] Wang C.C., Zhang W.J., Li H.D., Mao J.S., Guo C.Y., Ding R.Y., Wang Y., Fang L.P., Chen Z.L., Yang G.S. (2019). Analysis of volatile compounds in pears by HS-SPME-GC×GC-TOFMS. Molecules.

[21] Qian C.Y., Quan W.X., Xiang Z.M., Li C.C. (2019). Characterization of volatile compounds in four different *Rhododendron* Flowers by GC×GC-QTOFMS. Molecules.

[22] Gałuszka A., Konieczka P., Migaszewski Z.M., Namiesnik J. (2012). Analytical Eco-Scale for assessing the greenness of analytical procedures. Trends Anal Chem..

[23] Plotka-Wasylka J. (2018). A new tool for the evaluation of the analytical procedure: Green Analytical Procedure Index. Talanta.

[24] Xiang Z., Cai K., Liang G., Zhou S., Ge Y., Zhang J., Geng Z. (2014). Analysis of volatile flavour components in flue-cured tobacco by headspace solid-phase microextraction combined with GC×GC-TOFMS. Anal. Methods.

[25] Wajs A., Pranovich A., Reunanen M., Willfor S., Holmbom B. (2007). Headspace-SPME analysis of the sapwood and heartwood of *Picea abies*, *Pinus sylvestris* and *Larix decidua*. J. Essent. Oil..

[26] Hantao L.W., Toledo B.R., Ribeiro F.A., Pizetta M., Pierozzi C.G., Furtado E.L., Augustoa F. (2013). Comprehensive two-dimensional gas chromatography combined to multivariate data analysis for detection of disease-resistant clones of *Eucalyptus*. Talanta.

[27] Tajuddin S.N., Muhamad N.S., Yarmob M.A., Yusoff M.M. (2013). Characterization of the chemical constituents of agarwood oils from malaysia by comprehensive two-dimensional gas chromatography–time-of-flight mass spectrometry. Mendeleev Commun..

[28] Lonchamp J., Barry-Ryan C., Devereux M. (2009). Identification of volatile quality markers of ready-to-use lettuce and cabbage. Food Res. Int..

[29] Gasmalla M.A.A., Tessema H.A., Alahmed K., Hua X., Liao X., Yang R. (2017). Effect of different drying techniques on the volatile compounds, morphological characteristics and thermal stability of *Stevia rebaudiana* Bertoni leaf. Trop. J. Pharm. Res..

[30] Kumar R., Kumar R., Prakash O., Srivastava R.M., Pant A.K. (2019). GC-MS analysis of the hexane extract of *Limnophila indica* (L.) Druce, its total phenolics, in-vitro antioxidant, anti-inflammatory and antifeeding activity against *Spilosoma obliqua*. J. Entomol. Zool. Stud..

[31] Dekebo A., Kwon S.Y., Kim D.H., Jung C. (2018). Volatiles analysis of honey by gas chromatography-mass spectrometry (GC-MS): Comparison of SPME volatiles extraction methods. J. Apiculture.

[32] Caldeira M., Barros A.S., Bilelo M.J., Parada A., Câmara J.S., Rocha S.M. (2011). Profiling allergic asthma volatile metabolic patterns using a headspace-solid phase microextraction/gas chromatography based methodology. J. Chromatogr. A.

[33] Chandra D., Kohli G., Prasad K., Bisht G., Punetha V.D., Pandey H.K. (2017). Chemical composition of the essential oil of *Viola serpens* from Bageshwar (Shama), Uttarakhad, India. J. Med. Plants Res..

[34] Marneweck C., Jürgens A., Shrader A.M. (2017). Dung odours signal sex, age, territorial and oestrous state in white rhinos. Proc. R. Soc..

[35] Li S., Li P., Liu X., Luo L., Lin W. (2016). Bacterial dynamics and metabolite changes in solid-state acetic acid fermentation of Shanxi aged vinegar. Appl. Microbiol. Biotechnol..

[36] Oliveira R.C., Oi C.A., Vollet-Neto A., Wenseleers T. (2016). Intraspecific worker parasitism in the common wasp, Vespula vulgaris. Anim. Behav..

[37] Fernández M., Hospital X.F., Arias K., Hierro E. (2016). Application of pulsed light to sliced cheese: Effect on *Listeria* inactivation, sensory quality and volatile profile. Food Bioprocess Technol..

[38] Yang S. (2018). Study on the Volatile Components of Thirteen Medicinal Plants by SHS/GC-MS. Master’s Thesis.

[39] Jian H.J., Qiao F. (2018). Analysis of nutritional and volatile components in seeds and leaves of Synsepalum dulcificum. Stor. Proc..

[40] Yang Y., Zhang M., Yin H., Deng Y., Jiang Y., Yuan H., Dong C., Li J., Hua J., Wang J. (2018). Rapid profiling of volatile compounds in green teas using Micro-Chamber/Thermal Extractor combined with thermal desorption coupled to gas chromatography-mass spectrometry followed by multivariate statistical analysis. LWT-Food Sci. Technol..

[41] Domínguez R., Purriños L., Pérez-Santaescolástica C., Pateiro M., Barba F.J., Tomasevic I., Campagnol P.C.B., Lorenzo J.M. (2019). Characterization of volatile compounds of dry-cured meat products using HS-SPME-GC/MS technique. Food Anal. Method..

[42] Wang W., Huang X., Lin Y., Tang R., Guo Y. (2019). Analysis of volatile compounds in Guanyin tea stem. Chinese J. Trop. Crop..

[43] He Z., Fan Y., Xie X., Yuan G. (2012). GC-MS analysis of fragrance constituents from offcuts of eaglewood and Chinese Eaglewood in Guangdong. Adv. Mater. Res..

[44] Hussain A., Tian M.Y., He Y.R., Lei Y.Y. (2010). Differential fluctuation in virulence and VOC profiles among different cultures of entomopathogenic fungi. J. Invertebr. Pathol..

[45] Zhang Y., Li E., Wang C., Li Y., Liu X. (2012). *Ophiocordyceps sinensis*, the flagship fungus of China: Terminology, life strategy and ecology. Mycology.

[46] Simon C., Frati F., Beckenbach A., Crespi B., Liu H., Flook P. (1994). Evolution, weighting, and phylogenetic utility of mitochondrial gene sequences and a compilation of conserved polymerase chain reaction primers. Ann. Entomol. Soc. Am..

[47] Quan Q.M., Chen L.L., Wang X., Li S., Yang X.L., Zhu Y.G., Wang M., Cheng Z. (2014). Genetic diversity and distribution patterns of host insects of caterpillar fungus *Ophiocordyceps sinensis* in the Qinghai-Tibet plateau. PLoS ONE.

[48] Zhang Y.J., Zhang S., Li Y.L., Ma S.L., Wang C.S., Xiang M.C., Liu X.Z. (2014). Phylogeography and evolution of a fungal-insect association on the Tibetan plateau. Mol. Ecol..

[49] Silva É.A.S., Saboia G., Jorge N.C., Hoffmann C., Dos Santos Isaias R.M., Soares G.L.G., Zini C.A. (2017). Development of a HS-SPME-GC/MS protocol assisted by chemometric tools to study herbivore-induced volatiles in *Myrcia splendens*. Talanta.

[50] Wu Y., Xu J., He Y., Shi M., Han X., Li W., Zhang X., Wen X. (2019). Metabolic profiling of pitaya (*Hylocereus polyrhizus*) during fruit development and maturation. Molecules.

[51] Yan Y., Chena S., Niea Y., Xua Y. (2020). Characterization of volatile sulfur compounds in soy sauce aroma type Baijiu and changes during fermentation by GC×GC-TOFMS, organoleptic impact evaluation, and multivariate data analysis. Food Res. Int..

